# Considerations for spectroscopy of liquid-exfoliated 2D materials: emerging photoluminescence of *N*-methyl-2-pyrrolidone

**DOI:** 10.1038/s41598-017-17123-5

**Published:** 2017-12-01

**Authors:** Sean P. Ogilvie, Matthew J. Large, Giuseppe Fratta, Manuela Meloni, Ruben Canton-Vitoria, Nikos Tagmatarchis, Florian Massuyeau, Christopher P. Ewels, Alice A. K. King, Alan B. Dalton

**Affiliations:** 1 University of Sussex, Brighton, BN1 9RH United Kingdom; 2 Theoretical and Physical Chemistry Institute, National Hellenic Research Foundation, Athens, Greece; 3 Institut des Materiaux Jean Rouxel (IMN), Université de Nantes, Centre national de la recherche scientifique (CNRS), Nantes, France

## Abstract

*N*-methyl-2-pyrrolidone (NMP) has been shown to be the most effective solvent for liquid phase exfoliation and dispersion of a range of 2D materials including graphene, molybdenum disulphide (MoS_2_) and black phosphorus. However, NMP is also known to be susceptible to sonochemical degradation during exfoliation. We report that this degradation gives rise to strong visible photoluminescence of NMP. Sonochemical modification is shown to influence exfoliation of layered materials in NMP and the optical absorbance of the solvent in the dispersion. The emerging optical properties of the degraded solvent present challenges for spectroscopy of nanomaterial dispersions; most notably the possibility of observing solvent photoluminescence in the spectra of 2D materials such as MoS_2_, highlighting the need for stable solvents and exfoliation processes to minimise the influence of solvent degradation on the properties of liquid-exfoliated 2D materials.

## Introduction

*N*-methyl-2-pyrrolidone (NMP) is the prototypical solvent for dispersion of a range of carbon nanomaterials including fullerenes^1^, conjugated polymers^2^, nanotubes^3^ and graphene^4^. This general applicability is attributed to the matching of surface tension and Hansen solubility parameters with these materials^5–7^. This results in low enthalpy of mixing and allows individualised fullerenes, nanotubes and nanosheets to be dispersed at high concentration. Consequently, there is renewed interest in NMP for 2D materials beyond graphene such as molybdenum disulphide (MoS_2_) and black phosphorus. These layered materials require solvents which enable exfoliation from the bulk powder and stabilisation against restacking. While the surface tensions and Hansen parameters of these materials are difficult to measure directly and inherently dependent on the technique, MoS_2_, black phosphorus and many other layered materials can be easily exfoliated into NMP^8,9^.

While clearly an effective solvent, NMP is also known to be very susceptible to sonochemical polymerisation^10^ and degradation^11^ under the standard processing techniques for exfoliation of 2D materials. In addition, the yield and degree of exfoliation are also sensitive to the concentration of dissolved oxygen and water in the solvent^11–13^. Such sensitivities indicate that liquid phase exfoliation in NMP is not simply described by solubility parameter theory. The process involves chemical modification of the solvent, generating degradation and polymerisation products which influence the exfoliation process and results. While this may be desirable, the nature of these sonochemical processes can result in a wide variety of products which are difficult to characterise, and may be present as residual contaminants which influence the properties of the exfoliated nanosheets. The influence of degradation during exfoliation may lead to considerable variation of the performance of NMP as a solvent for a given nanomaterial, and potentially skewing of the measured surface tension and solubility parameters of the nanomaterial towards those of the pristine NMP^7^.

Furthermore, it has been noted that the optical properties of NMP are also influenced by its degradation, with yellowing of the solvent usually attributed to increased scattering of sonication products^10^. In addition, exfoliation yield and optical absorbance of NMP have also been acknowledged to vary with the age of the solvent^11^. Optical spectroscopy of NMP dispersions is further complicated, albeit rarely acknowledged in the literature on nanomaterials, by weak photoluminescence (PL) of the solvent^14,15^. We have observed the enhancement of this PL with degradation, with an increase in the overall intensity and the influence of a redshifted component in both aged, untreated samples and sonicated samples. The absorbance and photoluminescence of the degraded NMP present challenges for spectroscopy of nanomaterial dispersions, with the features still observed in as-received NMP and their influence exacerbated by the sonochemical degradation processes during exfoliation of the nanomaterial.

## Results

**Figure 1:**
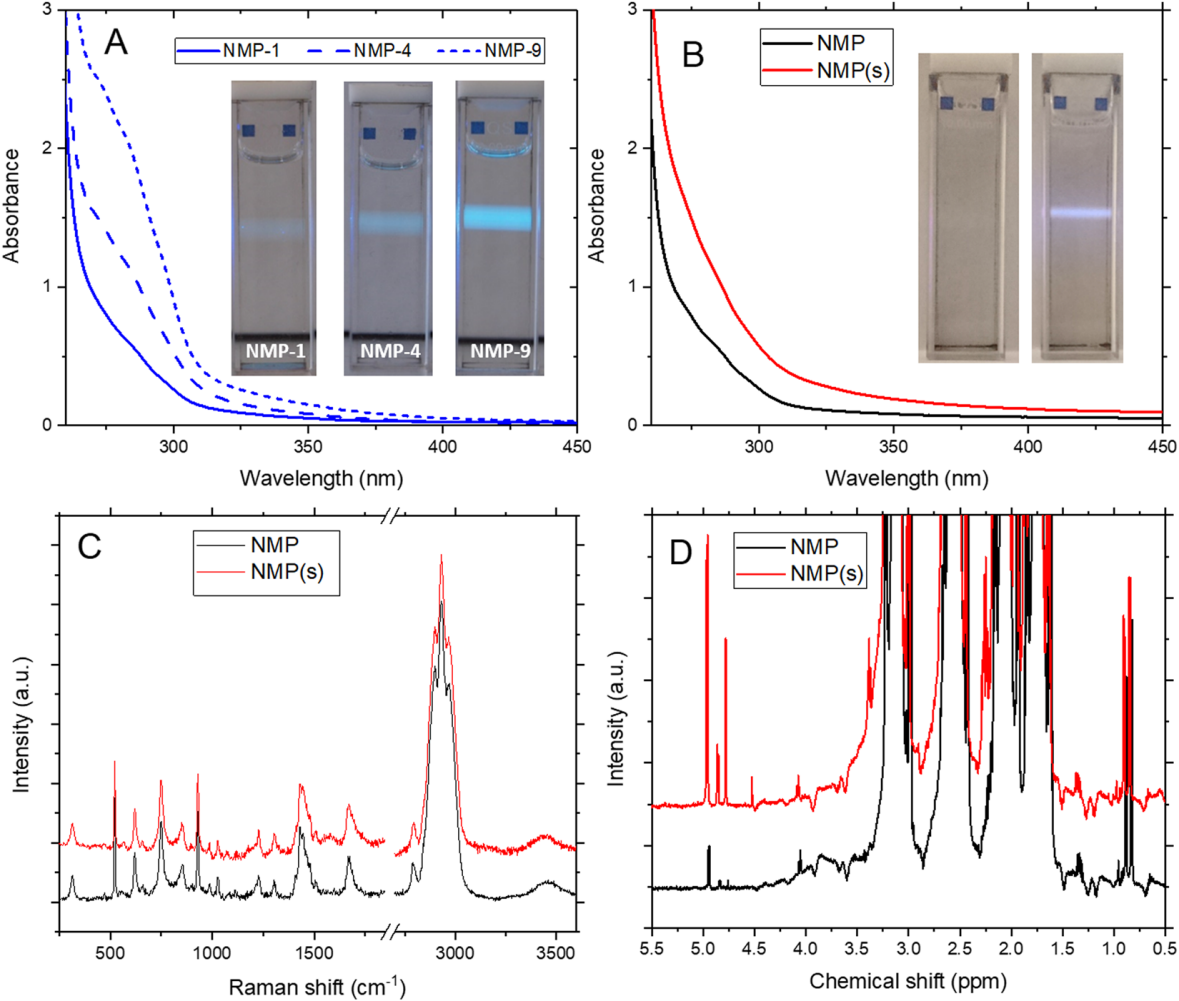
(**A**) UV-visible absorption spectra of NMP showing absorbance below 450 nm increasing with the age of the NMP, from NMP-1 to NMP-9. Inset: Photographs of NMP-1, NMP-4 and NMP-9 exhibiting strong blue-green photoluminescence under illumination with 405 nm laser pointer. (**B**) UV-visible absorption spectra for as-received NMP and sonicated NMP(s). (**C**) Raman spectra showing vibrational modes of NMP with no clear differences between as-received NMP and sonicated NMP(s). (**D**) ^1^H NMR spectra showing proton environments in NMP additional to those of the unmodified molecule with features corresponding to alkenyl species at a chemical shift of 5 ppm at around 0.1% by number relative to the unmodified NMP molecules, which increase in intensity from the as-received to the sonicated NMP(s).

The initial motivation for this study was the observation of visible yellowing of NMP samples with age as previously acknowledged by others^11^. Figure 1(A) shows UV-visible absorbance spectra for NMP samples stored in closed containers, in the dark and under ambient conditions for one, four and nine years. The samples were not externally treated in any way. Similar discolouration is also observed for sonicated NMP, hereafter referred to as NMP(s), produced from as-received NMP (HPLC grade, >99% purity). It is evident that such changes arise from increased optical absorption in the wavelength range below 450 nm.

Given that NMP has been reported to be weakly photoluminescent^14^, the samples were illuminated with a 405 nm laser. Under this excitation, all samples were observed to exhibit strong blue-green photoluminescence with PL intensity clearly increasing with both ageing and sonication of the NMP, as shown in the inset photographs in Fig. 1. While the differences in absorbance between the samples at this wavelength are small, the differences in PL intensity are significant and therefore not simply due to the increasing absorbance. This suggests that the increasing photoluminescence is due to the emergence of new species whose concentration or PL efficiency increases with degradation. Having made these observations in the aged samples, the sonicated samples were prepared to study degradation in a more controlled and practically-relevant manner. Data for the aged samples are presented in the supplementary information.

Raman spectroscopy was performed with laser excitation at 532 nm to compare the vibrational modes of the different samples and identify any new species present. These spectra show that NMP and NMP(s) have indistinguishable vibrational modes with fixed peak positions and intensities (Fig. 1(C)) which are also in excellent agreement with the solvent data sheet.

Subsequently, ^1^H nuclear magnetic resonance (NMR) spectroscopy was performed to identify any covalently-modified species which are present at trace levels and/or too similar in their vibrational modes to be detected by Raman spectroscopy. In addition to the expected proton peaks for the NMP, the NMR spectra show a number of peaks present at around 0.1% by number. The peaks with chemical shifts of around 5 ppm are attributed to alkenyl protons, suggesting the formation of RC=CH_2_ species, while the peaks around 0.9 ppm correspond to protons in a methyl group. These characteristic features are also observed in a previous study of sonochemical degradation of NMP which proposes a mechanism for formation of an enamine species (–N–C=C–) which undergoes polymerisation to form oligomeric nanoparticles^10^. The observation of similar features in the NMR spectra suggest that this mechanism could describe degradation due to both sonication and ageing. Furthermore, the presence of these features in the NMR spectra of all samples suggests that any degradation product is also present, at lower concentration, in the as-received NMP. Notably, the alkenyl and methyl protons associated with the previously-proposed mechanism of enamine formation and polymerization, are present and show downfield shifts relative to the as-received NMP. This is consistent with the downfield shifting of peaks expected due to deshielding by a nearby π-system, which could be a conjugated species which gives rise to the absorption and PL in both aged and sonicated NMP.

To determine whether the emergent absorption and PL are likely to be due to the pristine NMP or from a modified species, time-dependent density functional theory calculations were performed (see supplementary information). The unmodified NMP molecule was found to have optical transitions in the UV range of the spectrum only, with the primary absorption and emission features around 180 and 250 nm respectively, which strongly suggests that emerging properties in the visible range are due to a modified species. Using the previously-proposed enamine species as a starting point for degradation and polymerisation, the primary optical absorption redshifts by more than 40 nm. In addition, calculations show the emergence of a strong PL transition in the visible at 470 nm. While the modified species is likely to be more complex than the NMP derivative used, such calculations demonstrate that simple structural modifications can result in significant shifts and emergent transitions in the optical spectra of NMP.

**Figure 2:**
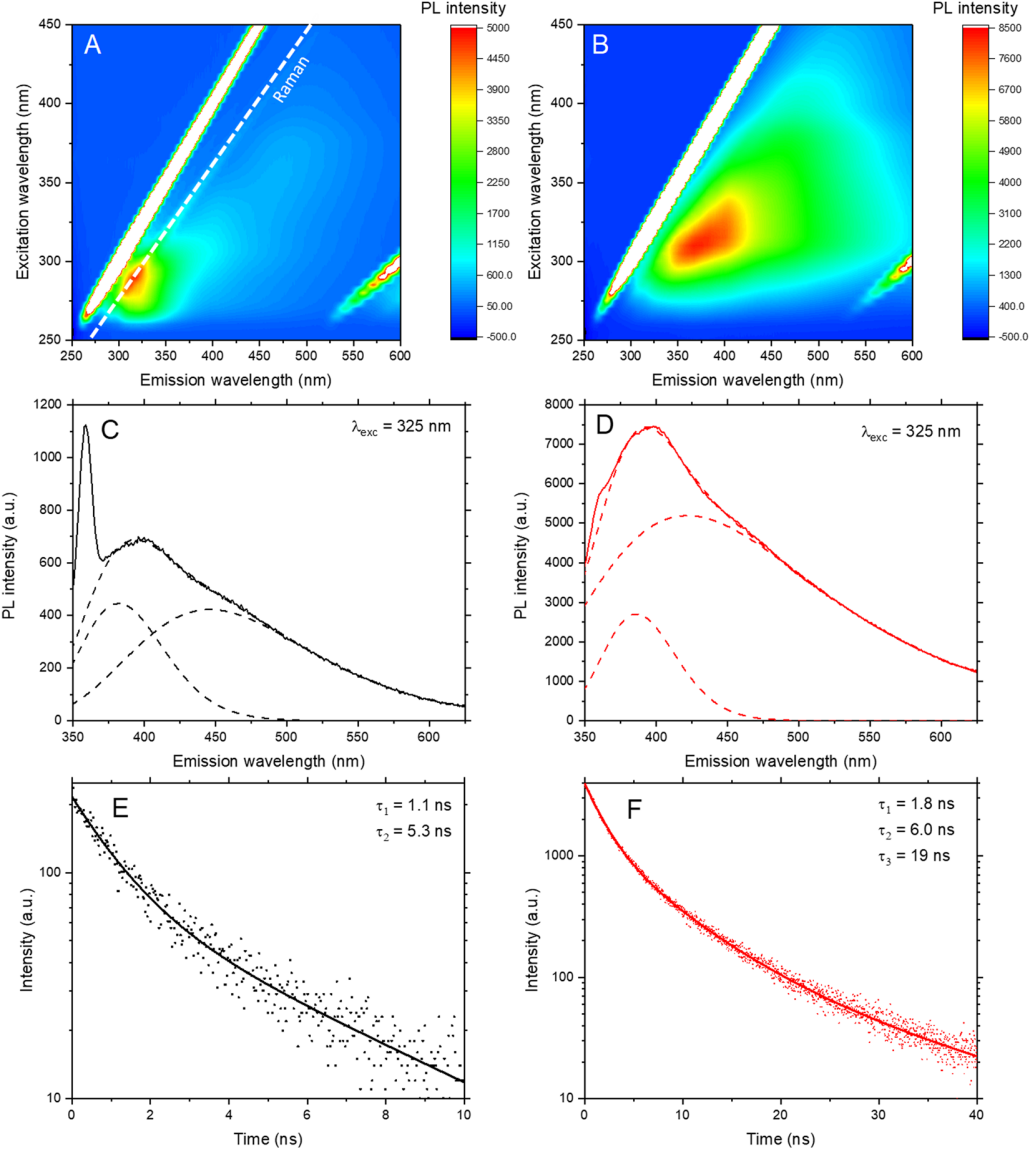
Photoluminescence excitation maps for (**A**) NMP and (**B**) NMP(s). PL spectra for peak emission with λ_exc_ = 325 nm for (**C**) NMP and (**D**) NMP(s), showing two component fitting with features at around 380 nm and 440 nm, the latter of which dominates PL emission in the NMP(s). Note the presence of an instrument-broadened Raman feature at 358 nm (~2900 cm^−1^), which has been excluded from the peak fitting. Time-resolved PL measurements of (**E**) NMP and (**F**) NMP(s) for emission at 400 nm under excitation at 336 nm, fitted as the sum of exponential components, whose time constants are shown inset, with increased lifetimes and an additional component present in NMP(s).

In order to ascertain the extent of the emission from the new species, PL spectroscopy was performed to obtain photoluminescence excitation (PLE) maps containing all excitation and emission spectra (Fig. 2(A and B)). These PLE maps indicate that NMP exhibits broad photoluminescence with peak emission around 400 nm under excitation at 325 nm. In addition, the spectroscopic changes after sonochemical degradation are illustrated by the changes in the intensity of the PL. For any given excitation wavelength, the peak PL intensity increases with the age of the NMP, resulting in the increasing brightness shown in the photographs in Fig. 1. The as-received NMP exhibits only very weak photoluminescence, with Raman features observed as prominently as the PL peaks. For NMP(s), the PL intensity is increased by an order of magnitude with appreciable emission across the whole visible spectrum.

The PL spectra for peak emission (λ_em_ = 400 nm, λ_exc_ = 325 nm) of NMP and NMP(s) are shown in Fig. 2(C and D) and can be fitted as the sum of two components, Gaussian in energy, at approximately 380 nm and 440 nm. Instrument-broadened Raman scattering of the excitation source (identified as the feature at ~2900 cm^−1^ in Fig. 1(C)) is present in the PL spectra at ~360 nm (Fig. 2(C)). The relevant data ranges were neglected from the curve fitting. These two PL components correspond to two emission species in the samples. When excited at lower energy than one or both of the species, as is common for photoluminescence measurements of 2D materials, the breadth of the features results in a redshifted contribution of that feature to the PL spectrum. This is illustrated by the significant PL intensity shown in the PLE maps for long wavelength excitation of NMP(s). The emission is dominated by the feature at 440 nm, which shows an apparent redshift with increasing excitation wavelength and could result in secondary excitation of the dispersed nanomaterial.

Time-resolved photoluminescence spectroscopy was performed to provide characterisation of the photoluminescence lifetimes of the species in the NMP samples. Figure 2(E and F) show time-resolved photoluminescence measurements with excitation wavelength of 336 nm at an emission wavelength of 400 nm, chosen to be as comparable as possible with the steady-state spectra shown above. It is evident that the total photoluminescence lifetime is significantly increased for NMP(s) compared with NMP. These time-correlated emission measurements can be fitted as the sum of exponential components, as shown in Fig. 2(E and F), where both samples were found to have a short-lived species with lifetime around 1 ns and a longer-lived species with lifetime around 5 ns. In addition, NMP(s) was found to have a third component with significantly greater lifetime of 19 ns. The relative abundances of the two shorter-lived species indicate that they likely correspond to the two components from the steady-state data, while the longer-lived species in NMP(s) is a much smaller contribution which was not identified in the steady-state fitting. In addition, the increase in the individual component lifetimes could suggest that the PL observed in as-received NMP is not due to the pristine NMP but due to the onset of ambient degradation. Greater delocalisation of the electron-hole pair in the excited state leads to the observed increase in the excited state lifetime. As such, the overall increase in lifetime with degradation is consistent with the formation of a larger, possibly polymerised, species.

## Discussion

**Figure 3:**
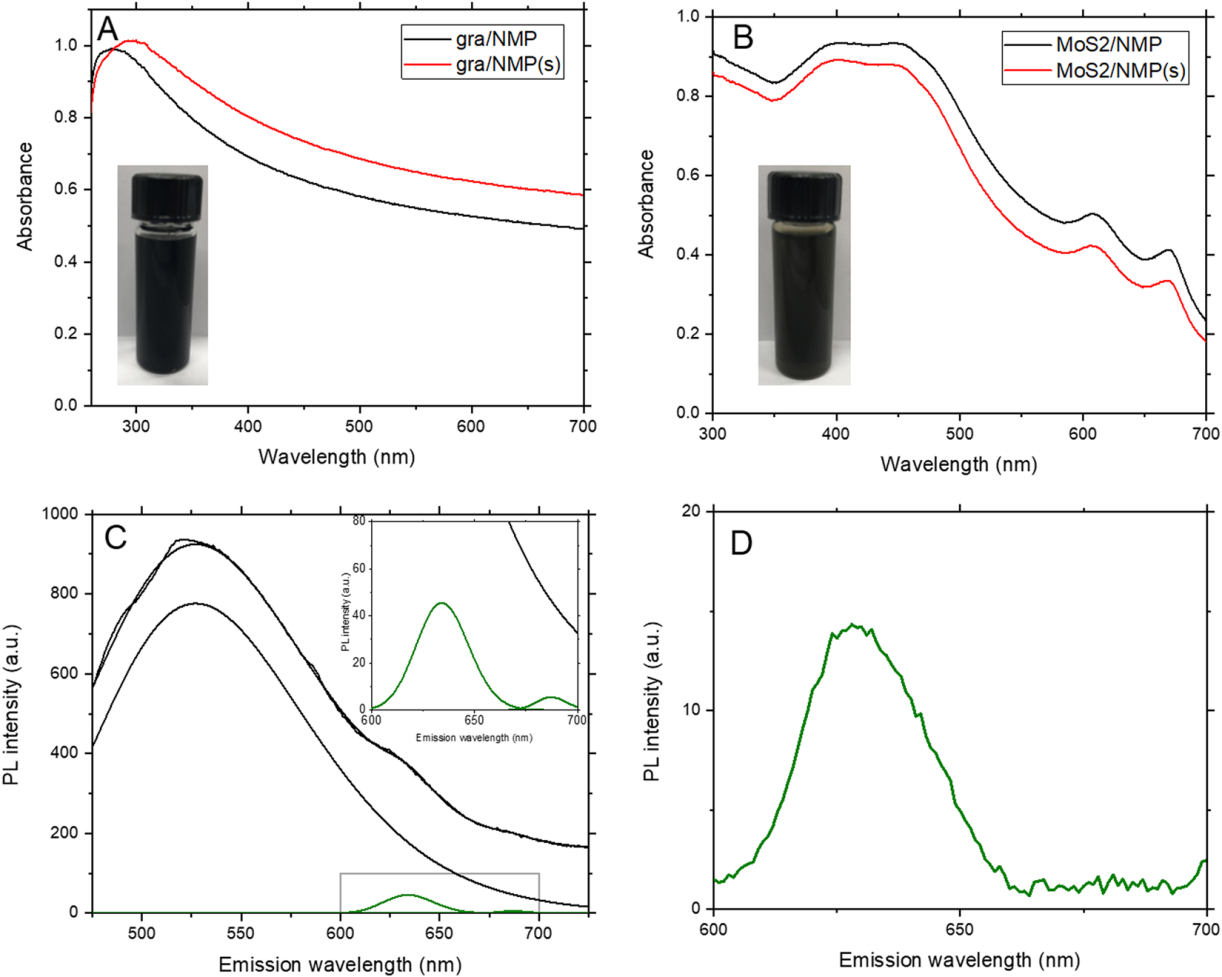
UV-visible absorption spectra for (**A**) graphene and (**B**) MoS_2_ exfoliated into NMP and NMP(s), showing significant increase in concentration for graphene exfoliated into NMP(s) and decrease in concentration for MoS_2_ exfoliated into NMP(s), compared with as-received NMP. (**C**) PL spectra for MoS_2_ showing PL of NMP considerably more prominent than, and overlapping with, PL of MoS_2_. Inset: expanded view of the same spectrum showing A and B exciton PL of MoS_2_. (**D**) PL spectrum of MoS_2_ after centrifugation and redispersion into surfactant and water, showing the absence of the broad background NMP PL.

It has been shown that sonication of NMP results in modification of both the chemical composition and the optical properties of the solvent. Sonochemical degradation has previously been shown to influence the behavior of NMP as a solvent for exfoliation and dispersion of nanomaterials^10,11^. The considerable changes observed with the relatively short sonication used to produce NMP(s) highlights the potential for the solvent degradation under typical exfoliation processes for 2D materials. In order to understand the influence of sonochemical degradation on liquid phase exfoliation of 2D materials, samples of graphene and MoS_2_ were exfoliated by sonication into as-received NMP and pre-sonicated NMP(s). UV-visible absorption spectra for the dispersions are shown in Fig. 3(A and B), which show opposing influences on exfoliation into pre-sonicated NMP(s). As previously demonstrated for carbon nanotubes^10^, the concentration of graphene dispersions is increased for exfoliation into NMP(s), as illustrated by the 10% increase in extinction. It is possible that this is due to solvation of the graphene nanosheets by a polymeric degradation product, similar to the polymer wrapping suggested for carbon nanotubes. While covalent modification of the nanomaterial has also been suggested as a potential mechanism, we note that liquid phase exfoliation in NMP has been shown to produce defect-free graphene^4^ and therefore anticipate that polymer wrapping is the more likely mechanism. By contrast, the concentration of MoS_2_ dispersions is decreased for exfoliation into NMP(s), suggesting that the production of degradation products and their interaction with the 2D materials may be sensitive to the chemical composition of the system. While the properties of these liquid-exfoliated dispersions are also likely to be influenced by the process itself, the differences observed demonstrate the sensitivity of the 2D materials to degradation of the solvent which will occur dynamically during exfoliation and is therefore inevitable for sonication-based exfoliation processes.

In addition, the influence of NMP degradation on the optical properties of the solvent present previously-unacknowledged challenges for spectroscopy of dispersions and solutions in NMP. Any dispersion produced by ultrasonication will be subject to degradation of the NMP, which further increases scattering in the solvent and the dispersion^10,11^. As such, it is not appropriate to use an untreated NMP sample as a reference for absorption spectroscopy as this will be chemically different from the sonicated NMP in the dispersion. However, if the reference sample is also sonicated, the ultrasonic cavitation field and thereby the sonochemical processes will differ significantly between the dispersion and the NMP reference sample. Importantly, this indicates that it is not possible to produce a reference sample of NMP for optical spectroscopy which is identical to the NMP in the nanomaterial dispersion. As such, the reference samples used for the spectra in Fig. 3(A and B) were the starting solvent NMP and NMP(s) respectively, which may no longer be representative of the solvent in the dispersion and the spectra therefore represent an upper bound on the absorption due to the 2D materials. This is clearly problematic for extinction-based measurements of concentration, size and thickness^16^ and highlights the need for alternative solvents and exfoliation processes.

Finally, the most significant influence of NMP degradation is in its use as a solvent for photoluminescence spectroscopy of nanomaterials, despite the solvent itself exhibiting strong and broad photoluminescence. Such nanomaterials include conjugated polymers^17^, carbon nanotubes^18^, transition metal dichalcogenides^19,20^ and black phosphorus^9,21^. For most of these materials, the photoluminescence is not in the same wavelength range as the NMP but the excitation wavelengths used could result in broad background luminescence from the solvent, although there is likely to be some quenching by the nanomaterial. This presents the possibility of the NMP emitting into the absorption band of the nanomaterial and providing an undesired longer wavelength excitation. For example, excitation of a dispersion of MoS_2_ or black phosphorus in NMP at 450 nm will result in PL emission from the NMP in the range 500–600 nm and further excitation of the dispersed nanosheets, potentially resulting in additional spectral features or influencing spectroscopic measurements of such materials. We note that this is particularly problematic for quantitative spectroscopic measurements such as that of monolayer fraction from PL and Raman spectroscopy^22^, which could be invalidated by secondary excitation of the MoS_2_ by PL emission from the NMP.

To illustrate the influence of NMP degradation, the PL spectrum for MoS_2_ exfoliated into NMP for excitation at 450 nm is shown in Fig. 3(C). MoS_2_ dispersions typically show weak PL since emission is only from monolayer nanosheets and self-absorbance reduces the outgoing signal. As such, the PL spectrum shows features at low intensity corresponding to A and B exciton PL at ~680 nm and ~625 nm respectively. The intense broad feature at ~530 nm is not due to the MoS_2_ but is instead due to the NMP, observed at longer wavelength under excitation at 450 nm. We note that this feature is likely to have been incorrectly attributed to MoS_2_ in previous studies^20^. To confirm this, the MoS_2_ dispersion was centrifuged at high speed to sediment the nanosheets out of the dispersion. The NMP was then discarded and the material was redispersed into aqueous surfactant solution in a manner similar to liquid cascade centrifugation^22^. Figure 3(D) shows the PL spectrum for the dispersion of MoS_2_ in surfactant and water. The broad background due to the feature at ~530 nm is no longer present, confirming that it is indeed PL from the degraded NMP, and the spectrum shows only the PL from the MoS_2_, albeit dominated by the B exciton due to self-absorbance by multilayers in the range of the A exciton. Together these spectra demonstrate the influence of NMP degradation on the PL spectra of liquid-exfoliated 2D materials and present a potential route, through sedimentation and redispersion, to transfer nanomaterials into fresh or different solvents to minimise the influence of solvent degradation on spectroscopy of 2D materials.

In conclusion, NMP, the most widely used solvent for 2D materials, has been shown to present significant challenges for liquid phase exfoliation due to its susceptibility to sonochemical degradation. It has been shown that NMP is even susceptible to such degradation with ageing under ambient conditions. Both ambient and sonochemical degradation result in chemically-similar products which give rise to strong visible photoluminescence of the NMP. In addition, this solvent degradation has been shown to influence the performance of NMP as a solvent for liquid phase exfoliation of 2D materials and perhaps even complicate the understanding of solubility parameters for solvent selection. Measurements of such dispersions are complicated by the increasing optical absorbance with solvent degradation and the nature of ultrasonic processing suggests that it is not possible to produce a sample of NMP as a reference sample for extinction spectroscopy, which is identical to the solvent in the dispersion. Furthermore, the emergence of photoluminescence of the degraded NMP influences spectroscopic measurements of photoluminescent materials in NMP, such as transitional metal dichalcogenides, with the PL spectrum of MoS_2_ in NMP shown to be dominated by the degraded solvent. These observations indicate that the use of NMP requires careful consideration for liquid phase exfoliation and optical spectroscopy of 2D materials.

## Methods

### Materials

NMP for the as-received and sonicated samples was purchased from Sigma Aldrich, product number 270458.

NMP for the aged samples was purchased from Sigma Aldrich and Fisher Scientific with the dates of receipt and product numbers as follows: NMP-9, 06/07/2007, Sigma 328634; NMP-4, 24/10/2012, Fisher M/5120/08; NMP-1, 01/10/2015, Sigma C112402. Molybdenum disulphide powder was purchased from Sigma Aldrich. Graphite powder was supplied by Zenyatta Ventures Ltd.

### Characterisation methods

UV-visible spectroscopy measurements were performed using a Shimadzu UV-2501PC spectrophotometer and Shimadzu UV-3600 Plus spectrophotometer using quartz cuvettes. Photoluminescence spectroscopy was performed using Cary Eclipse spectrophotometer and a Shimadzu RF-6000 spectrofluorometer. Raman spectra were acquired using an NT-MDT NTEGRA Spectra system with 473 nm laser excitation and Renishaw inVia system with 532 nm laser excitation. The photoluminescence background was subtracted using spine interpolation and the spectra were then normalized to the Raman mode at ~2900 cm^−1^. Time-resolved photoluminescence measurements were performed with a Horiba DeltaFlex TCSPC system with excitation at 336 nm, 349 nm and 409 nm using a 6 nm bandpass. 1H NMR spectroscopy was performed on a Varian VNMRS 600 spectrometer operating at a ^1^H frequency of 599.7 MHz.

### Sonication and liquid phase exfoliation

Sonochemical degradation of NMP was performed with a Sonics Vibra-Cell VCX130 ultrasonic probe. 20 mL of NMP was sonicated for 1 hour at 75% amplitude (~30 W power output), to produce the sample designated NMP(s). For the liquid-exfoliated graphene samples, graphite powder was added to 20 mL of NMP at an initial concentration of 25 mg/mL and sonicated for 1 hour at 75% amplitude. The sample was then centrifuged for 1 hour at 4000 g using a Thermo Scientific Sorvall Legend X1. For the liquid-exfoliated MoS_2_ samples, MoS_2_ powder was added to 20 mL of NMP at an initial concentration of 25 mg/mL. The supernatant was then discarded and the sediment was redispersed into another 20 mL of NMP. This was then sonicated for 1 hour at 50% amplitude with a pulse of 6 s on and 2 s off. The sample was then centrifuged for 1 hour at 2000 g, the sediment was discarded and the supernatant was collected. After the final centrifugation, all samples were left to stand overnight before characterisation. For the non-degraded samples, as-received NMP was used throughout the process. For the degraded samples, NMP(s) was used throughout the process.

## Electronic supplementary material

Supplementary Information
