# Supplementary material for: Considerations for spectroscopy of liquid-exfoliated 2D materials: emerging photoluminescence of N-methyl-2-pyrrolidone
Source: Sci Rep. 2017 Dec 1;7:16706. doi: 10.1038/s41598-017-17123-5 (PMC5711948; doi:10.1038/s41598-017-17123-5)
Supplement: Supplementary file 1 — Supplementary Information [file 41598_2017_17123_MOESM1_ESM.pdf]

Supplementary information

Considerations for spectroscopy of liquid-exfoliated 2D materials: emerging photoluminescence of N-methyl-2-pyrrolidone

Sean P. Ogilvie[1*], Matthew J. Large[1], Giuseppe Fratta[1], Manuela Meloni[1], Ruben Canton-Vitoria[2], Nikos Tagmatarchis[2], Florian Massuyeau[3], Christopher P. Ewels[3], Alice A. K. King[1] and Alan B. Dalton[1*]

[1] University of Sussex, Brighton, BN1 9RH, United Kingdom

[2] Theoretical and Physical Chemistry Institute, National Hellenic Research Foundation, Athens, Greece

[3] Institut des Materiaux Jean Rouxel (IMN), Universite de Nantes, Centre national de la recherche scientifique (CNRS), Nantes, France

S1. NMP degradation by ageing

S2. Time-dependent density functional theory calculations

S3. Atomic force microscopy of NMP-exfoliated graphene

S1. NMP degradation by ageing

The visible yellowing of NMP as a function of age was the initial motivation for this study, having observed this in samples of the solvent which had been purchased and left unused for several years. The discolouration is attributed to an increase in the absorption of the solvent in the blue region of the visible spectrum. Excitation of the aged NMP in this wavelength range was observed to result in visible photoluminescence of the solvent as shown in the main text. These aged samples were further characterised in parallel with the sonicated samples as shown in figure S1. While sonochemical degradation was studied as a well-controlled and practically-relevant process, characterisation of the chemical composition and optical properties indicates that ageing of NMP results in similar degradation and emphasises the susceptibility of NMP to such degradation even under ambient conditions.

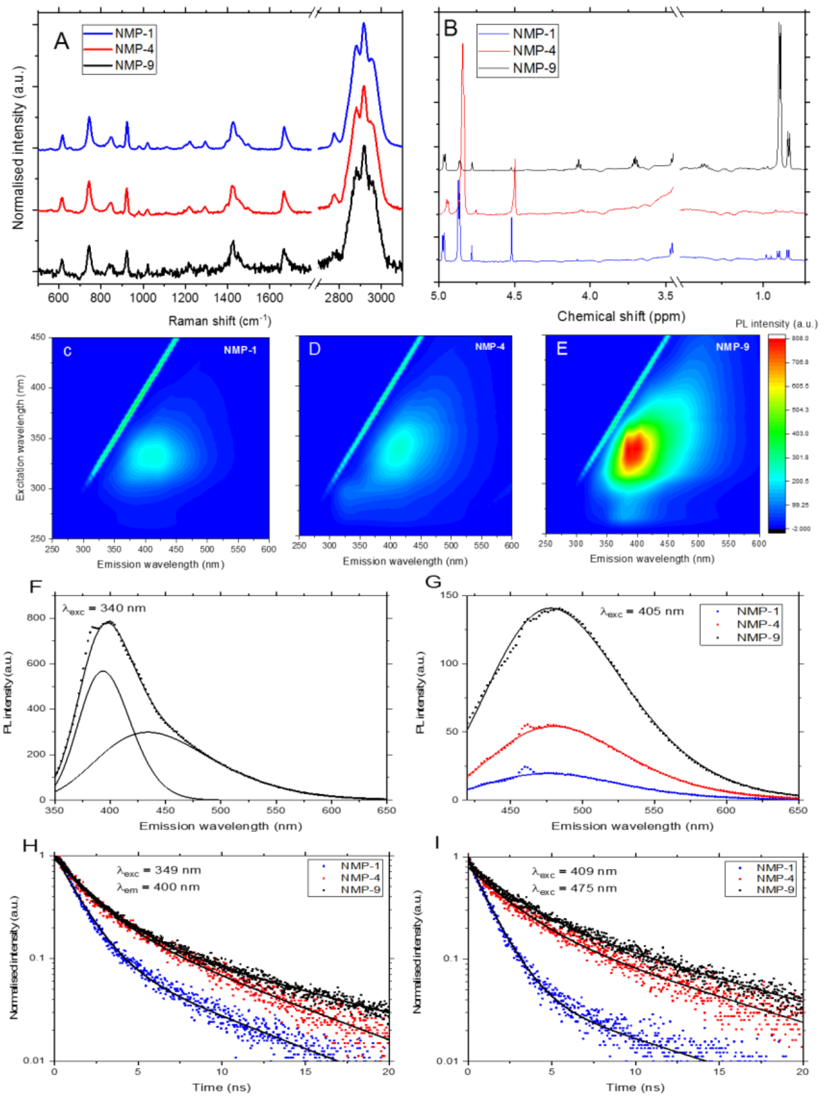

Figure S1. (a) Raman spectra of aged NMP showing no discernible differences between the samples indicating that the degradation products are present at very low concentration and/or similar vibrational frequencies to the pristine NMP. (b) ^{1}H NMR spectra of aged NMP with several peaks not associated with the unmodified NMP molecule, corresponding to alkyl and alkenyl peaks at around 0.9 and 4.8 ppm respectively, similar to those identified for the as-received and sonicated samples which suggests ageing could be described by a similar mechanism as sonochemical degradation. PLE maps showing the increasing PL intensity for (c) NMP-1, (d) NMP-4 and (e) NMP-9. (f) PL spectrum of NMP-1 for 340 nm excitation with two-component fitting confirming spectroscopically similar emission to that of NMP and NMP(s). (g) PL spectrum of aged NMP for 405 nm excitation with single component fitting

due to excitation of only the lower energy species in the samples. Note the intensity increase at the same excitation wavelength as for the photographs under laser excitation in the main text and the instrument-broadened Raman features at ~460 nm, which were neglected from the curve fitting. Time-resolved photoluminescence measurements of aged NMP for (h) 349 nm excitation and 400 nm emission and (i) 409 nm excitation and 475 nm emission, chosen to correspond to the wavelengths from the steady-state PL spectra above. Fitting as the sum of two exponential decays indicates that there is a short-lived species with lifetime around 1 ns and a longer-lived species with lifetime around 7 ns. The lifetimes are found to systematically increase as a function of age and the relative abundance of the longer-lived species also increases as function of age. These measurements are again consistent with the sonochemically-degraded samples and the proposed mechanism of enamine polymerisation into a conjugated species which gives rise to the emerging absorption and photoluminescence.

S2. Time-dependent density functional theory calculations

To determine the absorbance and photoluminescence characteristics of pristine NMP and an enamine-based NMP derivative proposed by Yau et al[1], time-dependent density functional theory calculations were performed. Table S1 lists the vertical UV-visible transitions calculated for NMP. While there is a very weak transition at 216.85 nm, the primary transition is in the UV at 180.14 nm, in good agreement with experiment. Using this excited state and re-optimising the geometry gives the calculated PL emission energy of 254.31 nm. This shows that pristine NMP is not responsible for the observed PL emission in the visible range.

Ring opening by cleavage of the N-CO bond and internal proton transfer has been proposed as the first step to subsequent NMP degradation and polymerisation, we therefore repeated the calculations with the NMP derivative (see Table S1). The primary absorption transition shows a ~40nm shift towards the visible. Additionally, in the calculated PL we see the emergence of strong signals in the visible range, at 474.36nm for the enamine derivative.

	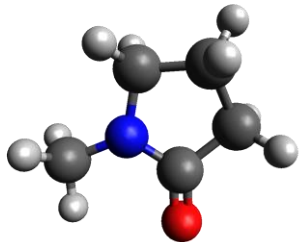		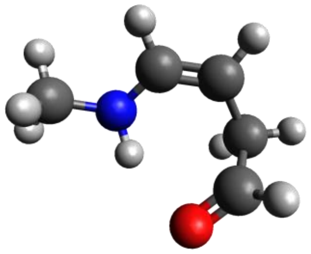	
	**NMP** (1-Methyl-2-pyrrolidone)		NMP enamine species (Yau et al., 2015)	
	Excitation Energy (nm)	Transition Moment *f*	Excitation Energy (nm)	Transition Moment *f*
	216.85	0.0012	328.56	0.0593
	195.70	0.0201	266.84	0.0166
	186.82	0.0070	219.37	0.0814
	180.14	***0.1882***	***213.59***	***0.1868***
	188.06	0.0287	**474.36**	**0.0380**
	**254.31**	**0.0929**	216.25	0.0141
	189.95	0.0022	219.93	0.0220
	361.63	0.0015	399.85	0.0004

Table S1. TD-DFT calculated absorption and emission energies at B3LYP/Def2TZVP-D3^{BJ} level. Italics indicates the principle excited state, used for structural optimization to obtain the PL emission. Bold indicates primary transition.

Methods

Structures were optimized using density functional theory using the global hybrid functional B3LYP, using the Def2TZVP basis[2-3] including the D3 Grimme dispersion correction with Becke-Johnson damping[4] as implemented in the Gaussian09D01 package[5]. Multiple initial structures were optimized and the lowest ground state structure chosen, with system energy minima confirmed via diagonalization of the Hessians. UV-Vis spectral information is obtained using time-dependent density functional theory (TD-DFT) using vertical transition energies. Photoluminescence vertical transitions are obtained

by optimizing the structure in the most intense vertical transition energy excited state (vibronic components are not taken into account).

S3. Atomic force microscopy of NMP-exfoliated graphene

While this study has mainly focussed on the influence of NMP degradation on spectroscopy of dispersions of 2D materials, it is acknowledged that it is possible (to some degree) to account for this by using reference samples or transferring into another solvent. Nevertheless, sonochemical modification of the solvent has been shown to influence exfoliation of 2D materials in NMP. As such, it is likely that NMP degradation products interact strongly with the materials and are adsorbed onto the nanosheet surface during exfoliation. Figure S2 shows at atomic force microscopy height image of NMP-exfoliated graphene with a considerable amount of residue present on the nanosheets after deposition and rinsing. The presence of this residue indicates that, even if the spectroscopic influence of NMP degradation can be accounted for, degradation products are likely to remain adsorbed onto exfoliated nanosheets and potentially influence performance in subsequent applications, particularly those reliant on inter-sheet tunnelling for conductivity.

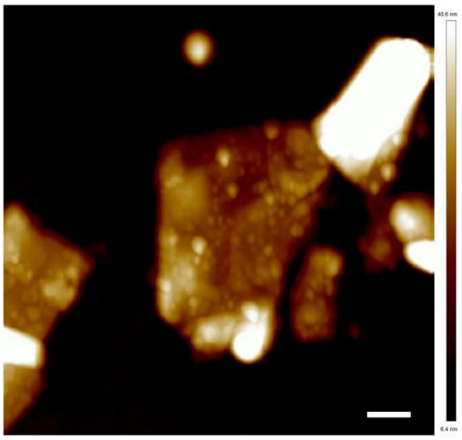

Figure S2. Atomic force microscopy height image of graphene nanosheet with adsorbed residue present after deposition and rinsing, illustrating the potential influence of NMP degradation products on subsequent applications of NMP-exfoliated 2D materials. Scale bar 200 nm.

References

1. Yau, H. C., Bayazit, M. K., Steinke, J. H. G. & Shaffer, M. S. P. Sonochemical degradation of N-methylpyrrolidone and its influence on single walled carbon nanotube dispersion. Chem. Commun. 51, 16621–16624 (2015).

2. F. Weigend and R. Ahlrichs, "Balanced basis sets of split valence, triple zeta valence and quadruple zeta valence quality for H to Rn: Design and assessment of accuracy," *Phys. Chem. Chem. Phys.*, **7** (2005) 3297-305.

3. F. Weigend, "Accurate Coulomb-fitting basis sets for H to Rn," *Phys. Chem. Chem. Phys.*, **8** (2006) 1057-65.

4. S. Grimme, S. Ehrlich and L. Goerigk, "Effect of the damping function in dispersion corrected density functional theory," *J. Comp. Chem*. **32** (2011) 1456-65.

5. Gaussian 09, Revision D.01, M. J. Frisch, G. W. Trucks, H. B. Schlegel, G. E. Scuseria, M. A. Robb, J. R. Cheeseman, G. Scalmani, V. Barone, B. Mennucci, G. A. Petersson, H. Nakatsuji, M. Caricato, X. Li, H. P. Hratchian, A. F. Izmaylov, J. Bloino, G. Zheng, J. L. Sonnenberg, M. Hada, M. Ehara, K. Toyota, R. Fukuda, J. Hasegawa, M. Ishida, T. Nakajima, Y. Honda, O. Kitao, H. Nakai, T. Vreven, J. A. Montgomery, Jr., J. E. Peralta, F. Ogliaro, M. Bearpark, J. J. Heyd, E. Brothers, K. N. Kudin, V. N. Staroverov, T. Keith, R. Kobayashi, J. Normand, K. Raghavachari, A. Rendell, J. C. Burant, S. S. Iyengar, J. Tomasi, M. Cossi, N. Rega, J. M. Millam, M. Klene, J. E. Knox, J. B. Cross, V. Bakken, C. Adamo, J. Jaramillo, R. Gomperts, R. E. Stratmann, O. Yazyev, A. J. Austin, R. Cammi, C. Pomelli, J. W. Ochterski, R. L. Martin, K. Morokuma, V. G. Zakrzewski, G. A. Voth, P. Salvador, J. J. Dannenberg, S. Dapprich, A. D. Daniels, O. Farkas, J. B. Foresman, J. V. Ortiz, J. Cioslowski, and D. J. Fox, Gaussian, Inc., Wallingford CT, 2013.
